# Health Service Utilization and Out-of-Pocket Expenditure Associated with the Continuum of Disability in Vietnam

**DOI:** 10.3390/ijerph18115657

**Published:** 2021-05-25

**Authors:** Liem Nguyen, John Tayu Lee, Emily S. G. Hulse, Minh Van Hoang, Giang Bao Kim, Duong Bach Le

**Affiliations:** 1 Institute of Population, Health and Development, Hanoi 117222, Vietnam; ntl1234@gmail.com; 2 Nossal Institute for Global Health, Melbourne School of Population and Global Health, University of Melbourne, Melbourne, VIC 3000, Australia; 3 Department of Primary Care and Public Health, Imperial College London, London SW7 2BX, UK; 4 Centre for Health Policy, Melbourne School of Population and Global Health, University of Melbourne, Parkville, VIC 3010, Australia; ehulse@student.unimelb.edu.au; 5 Hanoi University of Public Health, Hanoi 11909, Vietnam; hvm@huph.edu.vn; 6 Institute for Preventive Medicine and Public Health, Hanoi Medical University, Hanoi 116001, Vietnam; kimbaogiang@hmu.edu.vn; 7 The United Nations Population Fund (UNFPA), Hanoi 11109, Vietnam; dle@unfpa.org

**Keywords:** disability, continuum of disability, health utilization, out-of-pocket expenditure, Vietnam

## Abstract

Reducing the burden of disability is key priority in many countries where the population is aging rapidly. The relationships between disability, health expenditure and economic burden are complex, particularly when disability is recognized as a continuum rather than a dichotomous phenomenon. However, these complex relationships are not adequately addressed in national health policy and management plans in Vietnam. This paper examines the economic consequences of disability across its continuum or levels of severity. Two-part regression models were applied to assess the relationships between disability, health service use and the out-of-pocket expenditure. We found that Vietnamese adults with disabilities had multiple characteristics of vulnerability, e.g., older, less likely to be employed, lower education, and poorer than adults without disabilities. These characteristics are associated with poorer health and higher need of healthcare utilization but, after controlling for these factors, disability still had an independent association with higher health expenditure and greater economic burden at their household (*p* < 0.05). Our study provides empirical evidence of the economic burden associated across the continuum of disability in Vietnam. Decisive action is critical for protecting persons with disability from medical impoverishment, and such targeted interventions should include those with moderate disability rather than the current focus on severe disability.

## 1. Introduction

People with disability have complex health needs and are subject to a greater risk of financial catastrophe due to illness, especially in countries where healthcare is not free [1,2,3,4]. These studies also indicate that the impact of disability on health use and OOP health expenditure remain after controlling for socioeconomic factors, such as health insurance, poverty, employment, and education. The detrimental impact of healthcare costs on the poor, who are more at risk of acquiring a disability, has been coined by many as the “medical poverty trap” [5,6]. OOP health expenditure may force households to ‘cut their consumption of other minimum needs, trigger productive asset sales or high levels of debt, and lead to impoverishment’ [7] (p. 213). The World Bank substantiated this finding, maintaining that health costs are the most important precursor to poverty, after illiteracy and unemployment [8] (p. 1363).

People with disability in Vietnam are especially vulnerable to the impact of OOP expenditure. Recent statistics from the World Health Organization (WHO) show that OOP expenditure constitutes around 45% of total health expenditure in Vietnam, which is considerably higher than neighboring countries in the region [8,9]. Studies also have shown that in order to help paying medical bills, households sacrifice income and savings, take out high-interest loans, sell assets, borrow funds from family and friends or, in more extreme cases, withdraw children from schools to work [10]. Analyses by Van Minh et al. found that OOP health expenditure in Vietnam is linked to greater health inequity and medical impoverishment [11]. While the evidence in Vietnam is extremely limited given the rarity of data and interest in disability research, available evidence from Vietnam also shows that people with disability are greater consumers of healthcare services, experience higher economic burden than people without disabilities, and are at increased risk of healthcare induced poverty [12,13]. The heavy dependence of OOP expenditure to fund the health system is concerning, as growing evidence suggests user fees adversely affect health outcomes, especially for patients with a disability [11].

A major limitation of the current literature is the widespread use of the of dichotomous classification—with or without disability status. Over the past decade, the International Classification of Functioning, Disability, and Health (ICF) [14] has been increasingly used worldwide. Consequently, there has been a shift towards recognizing disability as a continuum rather than a dichotomous phenomenon. Another recommendation is reporting disability based on thresholds of difficulty in performing different activities [15,16,17,18]. This consideration of including severity in the measurement of disability is also increasingly adopted in Vietnam with the use of the Washington Group (WG) short set of questions [19] to operationalize the ICF framework in the 2006 Vietnam Living Standards Survey, the 2009 Population and Housing Census, and the 2011 Survey on Economic Cost of Disability-related Stigma in Vietnam. However, a very limited number of studies have used the ICF framework for in-depth analysis of disability or of the levels of disability in particular. Consequently, little is known beyond the dichotomous nature of disability or a small population of persons with a severe level of impairment, which has also been used in the Government’s policies and interventions to this point. To fill this important evidence gap, by adopting the ICF framework and the WG questions to take into account the multidimensional nature of the disability, this study aims to examine health service use and OOP expenditure associated with the continuum of disability in Vietnam. We hypothesize that persons with disabilities have higher OOP health expenditure and higher burden than persons without disabilities. In addition, significant differences may occur at not only a severe level but also other levels of disability.

## 2. Materials and Methods

### 2.1. Data

This paper uses data from the population-based 2011 Survey on Economic Costs of Disability-related Stigma in Vietnam. The study protocols and instruments were validated and piloted between June and July 2011 and the survey was carried out between July and September 2011 across eight provinces in Vietnam.

The survey used multistage stratified random sampling to collect information from adults. The study sample includes both the population with and without disability with an over-sample of the disabled population [12]. In the first stage, all provinces in Vietnam were stratified by (a) special metropolis (Hanoi and Ho Chi Minh City) or (b) non-special metropolis provinces (all other provinces). Both Hanoi and Ho Chi Minh City were selected. For the non-special metropolis stratum, one province from each of the six socioeconomic regions was randomly selected. In the second stage, two rural and four urban communes, i.e., sub-districts, were selected from each of the special metropolis provinces, and four rural and two urban communes were selected from each of the non-special metropolis provinces using probability proportional to size (PPS). In the third stage, two sub-communes were selected from each of the selected communes using the PPS. In the fourth stage, sampling frames, i.e., a list of adults with disability and a list of adults without disability, were constructed through a screening survey of all residents living in the selected sub-communes; then, 22 adults with disability and 22 adults without disability were randomly selected for interview. The response rate was 95% with 4093 individual participants across 3720 households completing the survey.

### 2.2. Variables

This study adopted the ICF framework and the WG questions to ascertain levels of disability [14,19]. The six WG questions ask individuals about difficulties they may have because of a health problem. These six domains of difficulty include seeing (even if wearing glasses), hearing (even if using a hearing aid), remembering or concentrating, walking or climbing stairs, self-caring (such as washing all over or dressing), and communicating (e.g., understanding or being understood by others). Response categories were—no difficulty, some difficulty, a lot of difficulty, and cannot do at all (or unable to do). In this paper, adults have been defined as people aged 15 and over. 

Adults without disability are defined as adults who had ‘no difficulty’ in any of the six domains of WG. Adults with mild disability are adults who reported ‘some difficulty’ in at least one of the six domains, and none of the domains were reported as ‘a lot of difficulty’ or ‘cannot do at all’. Adults with moderate disability are adults who reported ‘a lot of difficulty’ in at least one of the six domains, and none of the domains were reported as ‘cannot do at all’. Adults with severe disability are adults who reported ‘cannot do at all’ in at least one of the six domains. 

Respondents were asked about whether they had accessed healthcare—outpatient care during the preceding three months; inpatient care during the preceding twelve months; and self-treatment in the preceding three months. Inpatient care referred to medical care or treatment that was provided in any health facility and required at least one overnight stay. Outpatient care was defined as medical care and treatment that did not require an overnight stay. Self-treatment referred to an individual taking action on their own in managing their illness or condition. This included accessing ‘over-the-counter’ pharmacy consultations and medications, but excluded consultations with medical doctors. 

Respondents were also asked to identify their OOP expenditure related to these three categories of healthcare with expenditures, including direct costs for check-up fees tests, inpatient care, surgery, medication and indirect costs such as travelling, food and accommodation for both patients and caregivers. Costs paid or refunded through any health insurance were excluded. Monthly OOP expenditures were estimated under an assumption that health expenditure is equally distributed across the year. Monthly total OOP health expenditure was estimated by combining monthly expenditures for inpatient care, outpatient care, and self-treatment. 

To determine the catastrophic health expenditure at the household level, different thresholds were used within the range of 5–20% of total household income [20,21]. This study investigated health expenditure of individuals, given the unavailability of health expenditure data at the household level. OOP health expenditure was classified as imposing a high burden to the household, if the individual monthly OOP expenditure exceeded the threshold of monthly household income in the 5–20% range, reviewed at five percentage point intervals.

### 2.3. Statistical Approaches 

We assessed the health service use and OOP expenditure consequences associated with the continuum of disability using a series of two-part model—the first part predicted the likelihood of using healthcare services or self-treatment, while the second part estimated the amount of OOP expenditure among those who made any doctor visit or sought care. Multivariate logit regression was used in the first part of the model given the dichotomous nature of the dependent variable, i.e., using or not using healthcare. The second part of the model analyzed the OOP health expenditure with its distribution typically skewed to the right. As ordinary least squares (OLS) regression with a log-transformed expenditure model usually leads to biased estimates [3,22], a generalized linear model (GLM) with the log link and a gamma variance model specification was applied in our analysis. 

Our model controlled for other key demographic and socioeconomic variables and would be expected to have a strong association to OOP health expenditure as seen in previous studies; these variables include age, gender, education, employment, health insurance, homeownership, household income, household size, and rural/urban place of residence [1,2,23].

The survey data analysis procedures are adopted to address the complexity of survey design. Stata survey data commands, i.e., the svy prefix command, are used to properly handle the characteristics of survey design (i.e., sampling weight, cluster sampling, stratification) [24].

## 3. Results

### 3.1. Socio-Demographic and Socioeconomic Characteristics

The demographic and socioeconomic characteristics of the study population are presented in Table 1. Compared to adults without disability, adults with disability were generally older, had a lower educational attainment, less likely to be employed. However, adults with disability are more likely to work in their family business if economically active.

### 3.2. Health Service Use and Self-Treatment

Health service use by the disability continuum is shown in Table 2. A total of 71.8% of adults had either used healthcare services or self-treated in the preceding year (to be precise, 71.8% of adults either used inpatient care in the preceding twelve months, or outpatient care or self-treated in the preceding three months). Health service utilization was significantly higher among adults with disability compared to adults without disability. Patterns of health service use in adults with moderate disability compared to severe disability were similar. For example, approximately one-third of adults with moderate and severe disability and one-fourth of adults with mild disability had any inpatient visits last year; however, only one-tenth of adults without disabilities had any inpatient visits last year. Our data also revealed that self-treatment was the most common health seeking behavior among persons with disability. While approximately 50% of adults without disability chose self-treatment in the preceding three months, this figure was at least 60% higher in adults across all levels of disability.

### 3.3. OOP Expenditure by Continuum of Disability

Table 3 presents the average monthly OOP health expenditure for the following two groups: (1) those who had made any doctor visit, (2) the entire sample. Among those who had made any doctor visits, the largest proportion of OOP health expenditure was attributed to inpatient care, followed closely by outpatient care. OOP health expenditure for adults with severe disability was substantially higher than for other adults, particularly for inpatient and outpatient care. In our analysis that considered OOP healthcare expenditure from different sources (e.g., inpatient, outpatient, and self-treatment), our data revealed an exponential trend in the associations between levels of disability and OOP expenditure. The combined OOP healthcare cost for adults with mild disability was about 1.5 times higher than for adults without disability. Total OOP costs for adults with moderate disability were about two times higher than that of adults without disability, while the combined OOP costs for adults with severe disability was about four times higher than that of adults without disability (monthly total OOP expenditure was VND 256,268, 336,716, 439,135, and 1,030,276 in persons with no disability, mild disability, moderate disability, and severe disability, respectively).

The average OOP healthcare expenditure for the entire sample according to the level of disability was presented in the second part of Table 3. The results were broadly comparable with those in the above analysis using only those who had made any doctor visit. We found that a progressive increase in OOP healthcare costs according to the level of disability was also found (monthly total OOP expenditure was VND 161,439, 285,795, 395,374, and 906,396 in persons with no disability, mild disability, moderate disability, and severe disability, respectively).

Table 4 presents the proportion of OOP expenditure of household income by the continuum of disability. While OOP expenditure was equivalent to 8.2% of household income in our sample, this ranged from less than 5% among adults without disability to 9.6% among adults with mild disability and 35.2% for persons with severe disability. 

The proportion of adults with a high burden of OOP health expenditure by disability status using 5%, 10%, 15% and 20% thresholds was presented in Table 4. The proportion of adults with a high burden of OOP health expenditure was consistently greater among adults with higher levels of disability. 

### 3.4. Associations between by Severity of Disability and OOP Expenditures after Controlling for Other Covariates

Results from the two-part regression models are presented in Table 5. In the sample, 28% of adults had zero OOP health expenditure because they did not use any healthcare service or self-treat in the preceding three months of the survey (as specified in Table 2). A further 2.7% of the adults who had accessed healthcare services or treated themselves also had zero OOP expenditure as the costs were estimated to be covered or reimbursed by health insurance. 

The findings from the logistic regression models, predicting the likelihood of using healthcare services and/or self-treatment, revealed that the likelihood was significantly higher for adults with disability compared to adults without a disability. This significantly higher likelihood was found even after controlling for health insurance ownership status, household income, and other demographic and socioeconomic characteristics. It was found that adults with any level of disability were more likely than adults without disability to use healthcare services or self-treatment. The difference between adults with severe disability and adults without disability under the outpatient care expenditure regression model was not significant, and the difference for the self-treatment model was only marginally significant. 

Our results also show that adults with disability had greater OOP health expenditures than adults without disability after controlling for health insurance and other socioeconomic characteristics. It should be noted that significant and consistent differences were only found between adults with severe disability and adults without disability. Overall, adults with mild, moderate, and severe disability were associated with an additional 108,879, 181,682, and 353,283 VND per month, respectively, on healthcare than adults without a disability.

Findings from covariates show that being male, having health insurance, having a higher household income, and having a smaller household size were independently associated with higher OOP health expenditures while self-employed people were found to have lower OOP health expenditures. 

### 3.5. Disability and the Burden of OOP Health Expenditures after Controlling for Other Covariates

Analyses of the relationship between disability and the burden of OOP health expenditure after controlling for health insurance, household income and other socioeconomic characteristics indicated that adults with disability were significantly more likely to experience a high burden of OOP expenditure (see results in Table 6). For example, in comparison with people without a disability, those with a disability were more likely to incur OOP expenditure greater than 20% of the household income (AOR = 3.18, 4.87, 10.17 for persons with mild, moderate, and severe disability, respectively, *p* < 0.05). 

Findings from covariates indicated that being a female, older, not working for the State sector, and having a lower household income were independently associated with a higher likelihood of experiencing a high burden of OOP health expenditures.

## 4. Discussion

Our study found that the continuum of disability is associated with greater health service use and OOP expenditure in Vietnam. Disability is strongly associated with higher OOP health expenditure and economic burden of healthcare, independent of the type of healthcare sought, i.e., inpatient, outpatient, and self-treatment. This finding is consistent with an earlier study in Vietnam [5]. Similarly, another previous study focusing on children with disabilities found that a greater health expenditure and higher burden to households was observed compared to households without children with disabilities [7]. Consistent with previous studies, other socioeconomic factors, including gender, household size, household income, employment status, and health insurance status, had an independent effect on OOP health expenditures.

This study further identified that as the threshold to determine the high burden of OOP health expenditures increased, so did the risk of falling into the group with a high burden of OOP health expenditure of adults with disabilities compared to adults without disabilities. By examining patterns of health service use, we found that people with a disability were more likely to choose self-treatment for their health condition compared with those without any disability (approximately 65% vs. 50%). Overall, our study reveals a more comprehensive estimate of the association between levels of disability and economic consequences. These estimates can be used to inform economic models to predict the future burden disability in Vietnam by patients with different severity levels of disability. 

Consistent with findings both globally [18] and in Vietnam [5,12,13,25,26,27], this paper also showed that Vietnamese adults with disabilities were older, less likely to be employed, experienced worse education, and were poorer than the other adults. This study adds further evidence that there are challenges in employment for adults with disabilities [28]. Among those who are working, adults with disabilities are much more likely to work for their family or themselves than working for other people. While more than a third of working adults without disabilities worked for the State or private sector, only 10% of adults with moderate disabilities and 5% of adults with severe disability did the same. While none of the adults with severe disability were self-employed, the proportion of self-employed adults with moderate disability was similar to that of adults without disability, and it was highest among adults with mild disability. These differences in employment arrangements and participation in employment could be the consequences of self-stigma and environmental or contextual challenges for adults with disabilities [28]. Our results clearly show that OOP health expenditure and its burden was associated with severity of disability. These findings suggest that interventions that aim to alleviate the financial burden of disability should extend from the current focus of those with the most severe disability to those with moderate or even mild disability. 

There are several important limitations to this study. First, the cross-sectional study design limits causal interpretation of our findings. Further evidence is needed to assess the causal impact of the disability status on health service use and out-of-pocket expenditure using longitudinal data. It is also important to understand the potential pathways in which disability affects health service use. Second, non-OOP health expenditures, including costs paid or reimbursed by health insurance, were not collated. However, as this study has only reviewed OOP health expenditure, the impact of this limitation may be negligible. Third, although health insurance status is included, information regarding utilization was not collected, which may lead to an underestimation of the financial protection effect of health insurance on family finances [29]. In addition, the cost to the community more broadly has not been reviewed. While the survey drew on a population-based study, children (under the age of 15) and institutionalized people were excluded. As such, it is not representative of all people with disability in Vietnam, nor is it a full account of OOP health expenditure. 

## 5. Conclusions

Increased severity in disability status was associated with greater health service use and financial burden to patients and their households. The economic burden for people with disabilities was observed at a mild level of disability and increased exponentially along that continuum to severe disability. This supports the ‘new paradigm’ of disability as a complex social phenomenon requiring careful examination. Findings from this study provide evidence for policies and financial support to people with disabilities to avoid medical impoverishment due to their health conditions.

## Figures and Tables

**Table 1:** Demographic and socio-economic characteristics of the study population.

Variable	Level of Difficulty				All
	None	Mild	Moderate	Severe	
Mean age	36.4	49.6	59.5	58.1	42.4
(95% confidence interval)	(32.6–40.1)	(43.3–55.9)	(52.7–66.3)	(46.1–70.1)	(40.0–44.8)
Gender (%)					
Female	49.8%	53.6%	53.3%	51.0%	51.2%
Male	50.2%	46.4%	46.7%	49.0%	48.8%
Mean years of education	9.0	7.2	5.7	3.4	8.1
(95% CI)	(8.0–10.1)	(6.6–7.7)	(4.9–6.5)	(2.0–4.7)	(7.4–8.9)
Employment status (%)					
Not working	24.1%	30.4%	53.8%	92.2%	29.4%
Public sector	12.1%	4.3%	3.2%	– ^a^	8.9%
Private sector	15.9%	10.0%	1.7%	0.4%	12.8%
Family business	34.6%	39.4%	33.8%	7.4%	35.5%
Self-employed	13.3%	15.9%	7.5%	0%	13.4%
Health insurance (%)					
Never	29.1%	27.4%	22.6%	11.3%	27.8%
Previously, but not current insured	14.6%	13.5%	8.8%	7.0%	13.7%
Currently insured	56.3%	59.1%	68.5%	81.7%	58.5%
Mean household income preceding year (VND)	78,494,620	65,219,281	43,268,667	43,204,582	71,281,508
(95% confidence level)	(60,325,004–96,664,235)	(43,285,344–87,153,217)	(26,634,171–59,903,164)	(21,617,044–64,792,121)	(52,213,234–90,349,782)
Mean household size	4.2	4.0	3.5	3.9	4.1
(95% CI)	(3.9–4.5)	(3.3–4.6)	(2.4–4.5)	(2.5–5.2)	(3.6–4.6)
Place of residence (%)					
Rural	75.4%	78.6%	83.5%	88.9%	77.2%
Town	6.1%	9.0%	7.8%	3.5%	7.0%
City	18.5%	12.4%	8.7%	7.6%	15.8%
Province of residence (%)					
Hanoi	4.8%	1.7%	1.3%	2.4%	3.6%
Ho Chi Minh city	6.2%	2.8%	2.6%	4.5%	4.9%
Lang Son	23.8%	15.1%	7.2%	6.5%	19.7%
Thai Binh	21.2%	23.2%	32.0%	27.4%	22.8%
Quang Nam	14.2%	35.0%	35.1%	35.7%	22.2%
Kon Tum	1.9%	2.1%	4.1%	1.6%	2.1%
Dong Nai	11.1%	16.0%	9.5%	8.1%	12.3%
Vinh Long	16.8%	4.1%	8.2%	13.8%	12.4%
*n* (sample)	1855	1450	545	117	3967
*N* (population)	39,372,355	18,427,113	5,167,318	980,755	63,947,541

Notes: ^a^ Rounded to nil. Average 2011 exchange rate was USD 1 = VND 20,587 (www.ozforex.com, accessed on 1 April 2014); Minimum and maximum values of age, education, household income and household size could be provided upon request.

**Table 2:** Healthcare use (%).

Healthcare Use	Level of Difficulty				All
	None	Mild	Moderate	Severe	
Inpatient care	10.3	20.1	38.1	33.4	15.7
(95% CI)	(7.5–13.9)	(16.4–24.4)	(25.8–52.1)	(18.7–52.3)	(11.4–21.2)
Outpatient care	20.0	36.6	44.7	37.5	27.0
(95% CI)	(12.3–30.8)	(29.8–44.0)	(34.6–55.2)	(23.1–54.5)	(18.4–37.9)
Self-treatment	51.3	68.6	67.7	64.2	57.8
(95% CI)	(41.4–61.0)	(52.5–81.2)	(63.7–71.5)	(50.4–76.0)	(45.0–69.6)
Total healthcare usage	63.0	84.8	90.0	88.0	71.8
(95% CI)	(50.0–74.4)	(71.1–92.7)	(84.9–93.6)	(79.9–93.1)	(56.6–83.3)
*n*	1855	1450	545	117	3967
*N*	39,372,355	18,427,113	5,167,318	980,755	63,947,541

**Table 3:** Average monthly OOP healthcare expenditure (VND).

OOP Health Expenditure	Level of Difficulty				All
	None	Mild	Moderate	Severe	
**Users Only**					
Mean inpatient care	487,737	344,103	357,777	1,076,527	428,530
(95% CI)	(187,059–788,414)	(214,919–473,287)	(224,193–491,360)	(482,497–1,670,557)	(284,303–572,757)
*n*	209	289	193	40	731
*N*	4,039,515	3,696,446	1,968,590	327,471	10,032,022
Mean outpatient care	256,683	346,788	327,534	962,664	316,315
(95% CI)	(219,755–293,612)	(263,680–429,895)	(241,310–413,757)	(655,946–1,269,381)	(264,774–367,857)
*n*	380	515	236	33	1164
*N*	7,861,632	6,734,020	2,309,593	367,737	17,272,982
Mean self-treatment	117,249	129,059	164,767	289,643	128,699
(95% CI)	(68,979–165,518)	(85,646–172,473)	(103,405–226,129)	(146,489–432,797)	(92,577–164,821)
*n*	945	954	355	83	2337
*N*	20,166,164	12,623,757	3,465,556	629,789	36,885,266
Mean total healthcare usage	256,268	336,716	439,135	1,030,276	316,697
(95% CI)	(171,974–340,561)	(260,907–412,525)	(346,505–531,765)	(611,083–1,449,469)	(255,476–377,918)
*n*	1174	1192	489	103	2958
*N*	24,793,713	15,633,370	4,652,139	862,829	45,942,051
**All**					
Mean inpatient care	50,041	69,026	136,302	359,449	67,227
(95% CI)	(18,235–81,847)	(37,817–100,236)	(86,109–186,495)	(153,533–565,366)	(37,880–96,574)
Mean outpatient care	51,281	128,219	146,395	360,953	85,887
(95% CI)	(25,270–77,293)	(92,258–164,180)	(79,561–213,229)	(135,832–586,074)	(46,262–125,511)
Mean self-treatment	60,117	88,550	112,677	185,994	74,488
(95% CI)	(35,095–85,139)	(58,829–118,271)	(65,679–159,675)	(90,767–281,220)	(54,052–94,923)
Mean total healthcare usage	161,439	285,795	395,374	906,396	227,602
(95% CI)	(91,415–231,463)	(207,130–364,460)	(305,069–485,679)	(559,840–1,252,952)	(151,262–303,942)
*n*	1855	1450	545	117	3967
*N*	39,372,355	18,427,113	5,167,318	980,755	63,947,541

Note: Average 2011 exchange rate was USD 1 = VND 20,587 (www.ozforex.com, accessed on 1 April 2014).

**Table 4:** Burden of OOP health expenditure.

Burden of OOP	Level of Difficulty				All
	None	Mild	Moderate	Severe	
**Proportion of OOP Health Expenditure over HH Income**					
Mean	4.0	9.6	29.3	35.2	8.2
(95% CI)	(1.9–6.2)	(4.2–15.0)	(4.6–59.0)	(19.9–50.5)	(2.8–13.5)
*n*	1855	1450	545	117	3967
*N*	39,372,355	18,427,113	5,167,318	980,755	63,947,541
**Proportion of Adults Whose OOP Health Expenditure Exceed the Threshold of HH Income**					
5% threshold					
Mean	15.5	33.7	54.7	61.8	24.7
(95% CI)	(11.3–21.0)	(20.9–49.5)	(45.5–63.6)	(55.2–67.9)	(16.0–36.0)
10% threshold					
Mean	9.2	22.4	38.4	47.2	15.9
(95% CI)	(6.4–13.0)	(12.9–36.0)	(29.5–48.3)	(32.1–62.8)	(9.9–24.6)
15% threshold					
Mean	6.0	16.6	30.0	40.6	11.5
(95% CI)	(4.3–8.3)	(8.6–29.7)	(18.2–45.1)	(24.5–59.1)	(6.9–18.7)
20% threshold					
Mean	3.8	12.6	25.3	36.9	8.6
(95% CI)	(2.4–6.0)	(6.7–22.5)	(15.8–37.9)	(19.5–58.5)	(4.9–14.5)
*n*	1855	1450	545	117	3967
*N*	39,372,355	18,427,113	5,167,318	980,755	63,947,541

**Table 5:** Two-part model—relationship between disability and OOP health expenditures.

Level of Disability	Inpatient Care		Outpatient Care		Self-Treatment		Any Care	
Part 1: logit ^a^ (coefficients)								
None (*Ref.*)	0		0		0		0	
Mild	0.548	^***^	0.637	^**^	0.525	^***^	0.827	^***^
Moderate	1.207	^***^	0.759	^***^	0.531	^**^	1.149	^***^
Severe	0.856	^**^	0.366		0.513	^*^	1.000	^**^
Part 2: GLM ^b^ (coefficients)								
None (*Ref.*)	0		0		0		0	
Mild	0.059		0.333	^***^	0.167		0.311	^*^
Moderate	0.208		0.286		0.397	^*^	0.567	^**^
Severe	1.056	^**^	1.622	^***^	0.930	^**^	1.352	^***^
Predicted value combining logit and GLM (OOP expenditures in VND)								
None (*Ref.*)	0		0		0		0	
Mild	33,079	^*^	66,115	^***^	26,790	^***^	108,879	^***^
Moderate	78,107	^***^	68,903	^***^	44,047	^***^	181,682	^***^
Severe	116,449	^***^	164,857	^***^	83,196	^***^	353,283	^***^
*n*	3967		3967		3967		3967	
*N*	63,947,541		63,947,541		63,947,541		63,947,541	

Notes: *** *p* < 0.01, ** *p* < 0.05, * *p* < 0.10. All models include age, gender, completed education, employment, health insurance, (log) household income, household size, rural/urban place of residence, and province of residence. ^a^ Logit link is used. ^b^ Generalized linear models (GLM) with log links and gamma distribution are used. Survey data analyses and robust standard errors are used.

**Table 6:** Logistic regressions—Disability and high burden of OOP health expenditures.

Burden of OOP Expenditure	Odds Ratios		95% Confidence Interval
5% threshold			
None (Ref.)	1		
Mild	1.99	^***^	1.27–3.12
Moderate	3.32	^***^	2.77–3.98
Severe	4.99	^***^	2.25–11.04
10% threshold			
None (Ref.)	1		
Mild	2.14	^**^	1.24–3.69
Moderate	3.00	^***^	2.00–4.50
Severe	4.79	^***^	1.86–12.36
15% threshold			
None (Ref.)	1		1
Mild	2.58	^**^	1.31–5.11
Moderate	3.63	^***^	1.68–7.82
Severe	6.78	^***^	2.39–19.19
20% threshold			
None (Ref.)	1		1
Mild	3.18	^***^	1.68–6.03
Moderate	4.87	^***^	2.08–11.40
Severe	10.17	^***^	3.01–34.40
*n*	3967		3967
*N*	63,947,541		63,947,541

Notes: *** *p* < 0.01, ** *p* < 0.05. All models include age, gender, education completed, working status, health status, health insurance, (log) household income, household size, rural/urban place of residence, and province of residence. Survey data analyses and robust standard errors are used.

## Data Availability

Data supporting reported results can be obtained by request to the Institute of Social Development Studies.
